# The Unchanging Latency of Transcranial Motor-Evoked Potentials Among Various Age Groups

**DOI:** 10.7759/cureus.74749

**Published:** 2024-11-29

**Authors:** Tania Talwar, Uditi Gupta, Sreya Konusu, Megha Bir, Hanjabam Barun Sharma, Ashok Kumar Jaryal

**Affiliations:** 1 Physiology, All India Institute of Medical Sciences, New Delhi, New Delhi, IND; 2 Physiology, All India Institute of Medical Sciences, Jammu, Jammu, IND; 3 Physiology, Institute of Medical Sciences, Banaras Hindu University (BHU), Varanasi, IND

**Keywords:** age, bodily dimensions, intraoperative neuromonitoring, latency, motor proficiency, motor response, nerve conduction study (ncs), tcmep, transcranial motor evoked potential

## Abstract

Background

Human growth and development involve significant changes in bodily dimensions, yet motor learning appears to remain stable throughout life. This study investigates whether adjustments in motor velocity take place as individuals age by examining the latency of transcranial motor-evoked potentials (TcMEPs) across different age groups.

Methods

Data were collected from 100 patients who underwent surgery with intraoperative neuromonitoring at the All India Institute of Medical Sciences, New Delhi, between January 1, 2019, and January 1, 2020. TcMEP recordings were analyzed for 7 commonly monitored muscles across 7 distinct age groups: under 10 years, 10-19 years, 20-29 years, 30-39 years, 40-49 years, 50-59 years, and over 60 years.

Results

The analysis revealed no significant differences in motor-evoked potential (MEP) latency across the age groups, indicating that motor response latency remains stable despite the physical changes that occur with aging.

Conclusion

These findings enhance our understanding of motor learning, suggesting that motor response latency does not necessitate changes with age, highlighting the consistency of motor function over the human lifespan.

## Introduction

Human growth and development involve dynamic changes in bodily dimensions, especially during growing years of life, yet motor learning appears to remain consistent despite these changes. This observation suggests that as individuals grow, adjustments must be made to maintain motor proficiency, often manifesting as increased velocity in movements to achieve the same level of performance as evidenced by Salthouse, Feldman et al., and Desmedt et al. [1-3]. While theoretical and animal models by Swärd et al. and Raminsky et al. propose that velocity adjustments are necessary to compensate for changing dimensions [4,5], there is a lack of empirical studies examining this phenomenon in humans. Intraoperative neuromonitoring offers a unique opportunity to bridge this gap in knowledge, providing real-time data on neural and motor functions during surgical procedures and presenting a chance to observe how variations in bodily dimensions affect motor performance under controlled conditions. By leveraging this approach, our study aims to investigate how growth-induced changes in bodily dimensions influence motor velocity, providing insights that have been only captured through other methods like electrodiagnostic nerve conduction studies (Rivner et al. [6]), motor-evoked potentials, and central motor conduction using transcranial magnetic stimulation (Booth et al. [7]), and F-waves by Imajo et al. [8].

## Materials and methods

Study design and patient selection

This study was a retrospective analysis utilizing data from patients who underwent surgical procedures with neuromonitoring using transcranial motor-evoked potentials (TcMEPs) at the All India Institute of Medical Sciences, New Delhi, between January 1, 2019, and January 1, 2020. Inclusion criteria focused on patients of various age groups undergoing surgery where neuromonitoring was implemented. Exclusion criteria encompassed the following conditions, which are known to affect motor conduction: 1) neurological or neuromuscular transmission disorders, including weakness of upper and/or lower limbs; 2) history or presence of epilepsy, moderate-to-severe traumatic head injury, previous cranial or spinal surgery, stroke, acute/advanced/chronic uncompensated medical illnesses (e.g., diabetes, hypertension, thyroid disorders), or drug abuse; 3) current treatment with neuroactive drugs or other medications affecting cortical excitability. The study received ethical clearance from the Institute Ethics Committee for Post Graduate Research at All India Institute of Medical Sciences (Ref. No.: IECPG - 199/27.03.2019, OT-13/27.01.2021).

**Figure 1:**
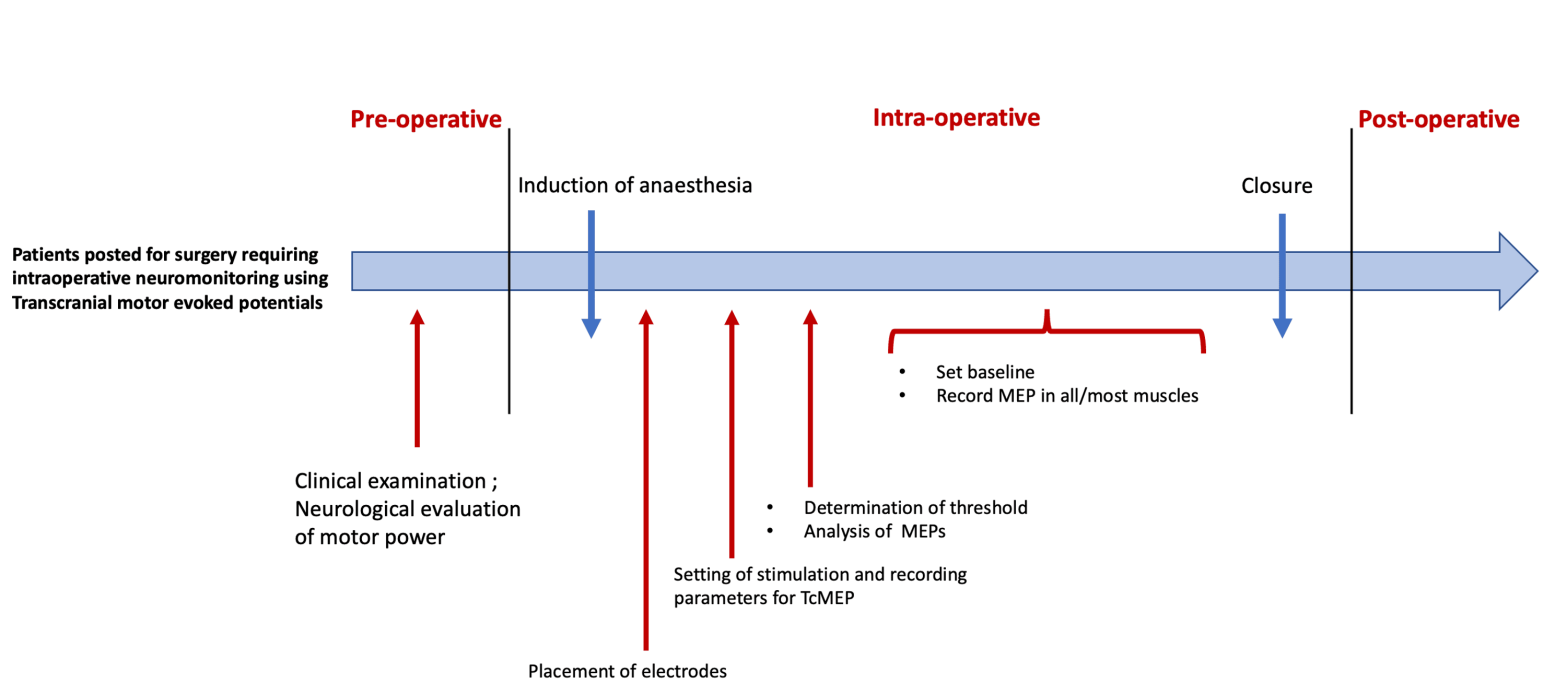
Schematic representation of the procedure in the operation theater MEP: motor-evoked potential; TcMEP: transcranial motor-evoked potential

The neuromonitoring protocol for each patient was meticulously followed, spanning from pre-surgery assessment to post-surgery follow-up. A detailed protocol outline is illustrated in Figure 1.

Preoperative assessment

Prior to surgery, demographic and clinical data, including age, gender, and diagnosis were collected. A comprehensive medical history and physical examination were conducted for all patients. Neurological evaluations were performed to assess motor power using the Medical Research Council (MRC) scale (Medical Research Council, UK).

Intraoperative neuromonitoring

During surgery, total intravenous anesthesia was administered using propofol (100-150 µg/kg/min) and fentanyl (1 µg/kg/hr). A short-acting muscle relaxant was administered solely during induction. The target bispectral Index (BIS) range for depth of anesthesia was 40-60, and the core temperature was maintained above 36 °C. After induction, dual twisted needle electrodes were positioned for motor-evoked potential (MEP) recording. The active electrode was placed perpendicular to the muscle belly, while the reference electrode was positioned approximately 5 centimeters away in the subcutaneous plane and secured with micropore tape. Electrode impedance was checked, and replacement was done if the impedance exceeded 15 kΩ.

MEP recordings were performed from various muscles depending on the location of the lesion. For brain lesions, contralateral muscles were monitored with ipsilateral controls. In the case of spinal cord lesions, both bilateral muscle recordings were utilized, with muscles above the surgical site serving as controls and those below the site being used for intraoperative monitoring. The specific muscles used for MEP recordings varied depending on the surgical requirements, patient positioning, and available time, but typically included: brachioradialis (BR), abductor pollicis brevis (APB), quadriceps femoris (QD), tibialis anterior (TA), extensor hallucis longus (EHL), abductor hallucis (AH), and anal sphincter (AS). Other muscles included (not for the purpose of monitoring) were the frontalis, orbicularis oculi, orbicularis oris, mentalis, trapezius, deltoid, biceps, triceps, flexor carpi radialis, abductor digiti minimi, rectus abdominis, biceps femoris, and gastrocnemius. For this research, data were collected from as many muscles as possible, including the specific muscles of interest that were directly monitored for neurophysiological support during the surgery. Thresholds were determined for each muscle where feasible, and latency data was also collected. However, data for threshold and latency were not available for all 100 patients across all muscles. Specifically, the data presented in this study primarily reflect the seven most frequently monitored muscles, for which full data (threshold and latency) were consistently available.

Following electrode placement, patient positioning (prone, supine, or lateral) was adjusted according to the surgical approach. Impedance levels were checked and re-evaluated as necessary, with electrodes being repositioned to ensure optimal recording conditions.

The stimulation parameters were not uniform across all muscles. A range of stimulation parameters was employed to determine the threshold and latency for each muscle for this research, but not for final monitoring. Transcranial electrical stimulation was delivered using corkscrew electrodes positioned at C3' and C4' (1 cm anterior to C3 and C4) according to the international 10-20 electrode placement system. Motor cortex stimulation was performed using fast charge, consisting of a train of 2-8 biphasic pulses with a duration of 50-75 µs, an inter-stimulus interval (ISI) of 2-4 ms, and a frequency of 250-500 Hz. Threshold stimulation was determined by gradually increasing voltage from 50V with increments of 25V until a measurable MEP response was obtained in most muscles. This threshold was used for subsequent monitoring. MEPs were recorded in a 100 ms window with a band-pass filter set to 30-3000 Hz, utilizing the NIM-Eclipse E4 System (Medtronic, Minneapolis, MN, US). Data were tabulated and analyzed using Microsoft Excel (Microsoft Corporation, Redmond, WA, US) for the study.

Postoperative follow-up and data analysis

**Figure 2:**
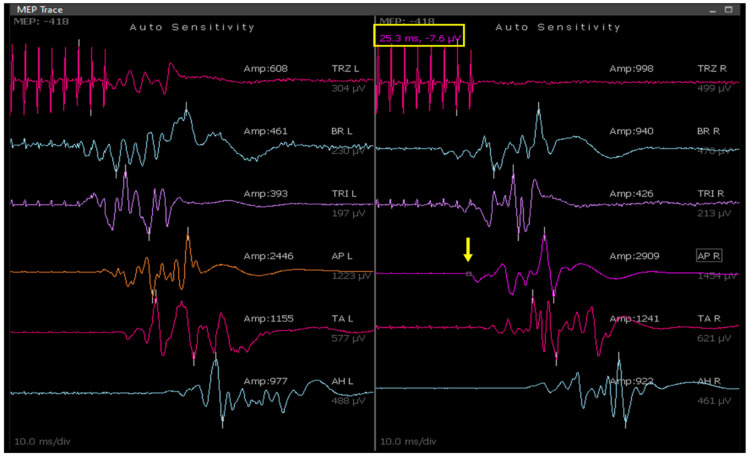
MEP record showing the latency of the right abductor pollicis brevis (APB) muscle, indicated by yellow arrow The corresponding value of the latency of response is seen at the top of the figure in the yellow box. MEP: motor-evoked potential

Postoperatively, patients were assessed for motor function, and recorded data were analyzed to quantify and compare the latencies of MEP responses in muscles across different age groups. Average latencies for individual muscles were determined. The latency of response was measured from stimulation to the appearance of motor-evoked potentials (Figure 2).

Latency values for each muscle on both sides were measured, and mean latencies were calculated for each patient. Patients were categorized into age groups: <10 years, 10-19 years, 20-29 years, 30-39 years, 40-49 years, 50-59 years, and >60 years.

Statistical analysis of stimulation and recording data was performed using one-way analysis of variance (ANOVA) on GraphPad Prism v9.0.2 (GraphPad Software, Inc., California, USA) to assess the effects of age on latency.

## Results

A total of 146 patient records were screened, and 100 patients met the inclusion criteria for this study. The exclusion of 46 patients was due to non-compliance with the exclusion criteria or inadequate MEP records.

Patients were organized into the specified age groups (Table 1).

**Table 1:** Patient characteristics

Patient Characteristics						
	Female		Male		Total	
Age Group	N	%	N	%	N	%
<10	10	10%	13	13%	23	23%
10-19	6	6%	13	13%	19	19%
20-29	7	7%	8	8%	15	15%
30-39	7	7%	16	16%	23	23%
40-49	1	1%	6	6%	7	7%
50-59	3	3%	4	4%	7	7%
>60	2	2%	4	4%	6	6%
Total	36	36%	64	64%	100	100%

Latency for each muscle on both sides was measured, and the average latencies (in milliseconds) for seven commonly monitored muscles were assessed across various age groups: under 10 years, 10-19 years, 20-29 years, 30-39 years, 40-49 years, 50-59 years, and over 60 years (Table 2).

**Table 2:** Average latency (milliseconds) for BR, APB, QD, TA, EHL, AH, and AS for all age groups BR, brachioradialis; APB, abductor pollicis brevis; QD, quadriceps; Ta, tibialis anterior; EHL, extensor hallucis longus; AH, abductor hallucis; AS, anal sphincter

Age Groups (years)	BR	APB	QD	TA	EHL	AH	AS
<10	23.2	26.7	27.8	34.6	34.9	41.4	38.2
10-19	22	26.9	22.8	34.3	31	42.7	27
20-29	24.2	24.4	29.2	34.3	35.7	48.7	35
30-39	21.4	27.5	29.4	34.1	35.5	44.4	32.2
40-49	21.4	28	30.4	33.6	36.4	44.2	34.6
50-59	20.3	25.7	26.9	31.3	38.4	44.9	25.4
>60	18.3	27.3	22.9	35.4	31.6	47.4	-

**Figure 3:**
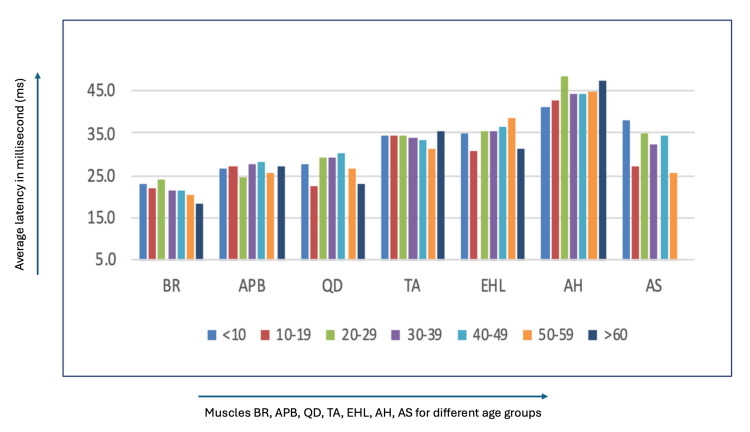
Average latency in milliseconds (ms) for BR, APB, QD, TA, EHL, AH, and AS for each age group On the X-axis: Muscles BR, brachioradialis; APB, abductor pollicis brevis; QD, quadriceps; Ta, tibialis anterior; EHL, extensor hallucis longus; AH, abductor hallucis; AS, anal sphincter Age groups: <10 years, 10-19 years, 20-29 years, 30-39 years, 40-49 years, 50-59 years, >60 years On the Y-axis: Average latency in milliseconds (ms)

Figure 3 illustrates the average latency for each age group using a grouped bar chart.

Statistical analysis revealed no significant differences in latency with age, as indicated by statistically insignificant P-values (Table 3).

**Table 3:** Comparison (one-way ANOVA) of latencies for BR, APB, QD, TA, EHL, AH, and AS for all age groups p-value ≤0.05 = statistically significant BR, brachioradialis; APB, abductor pollicis brevis; QD, quadriceps; TA, tibialis anterior; EHL, extensor hallucis longus; AH, abductor hallucis; AS, anal sphincter; ANOVA: analysis of variance

Muscles	P-value	R-squared
BR	0.8960	0.04072
APB	0.4611	0.07102
QD	0.5390	0.08758
TA	0.9524	0.01912
EHL	0.2833	0.2579
AH	0.1608	0.1041
AS	0.6959	0.1044

**Figure 4:**
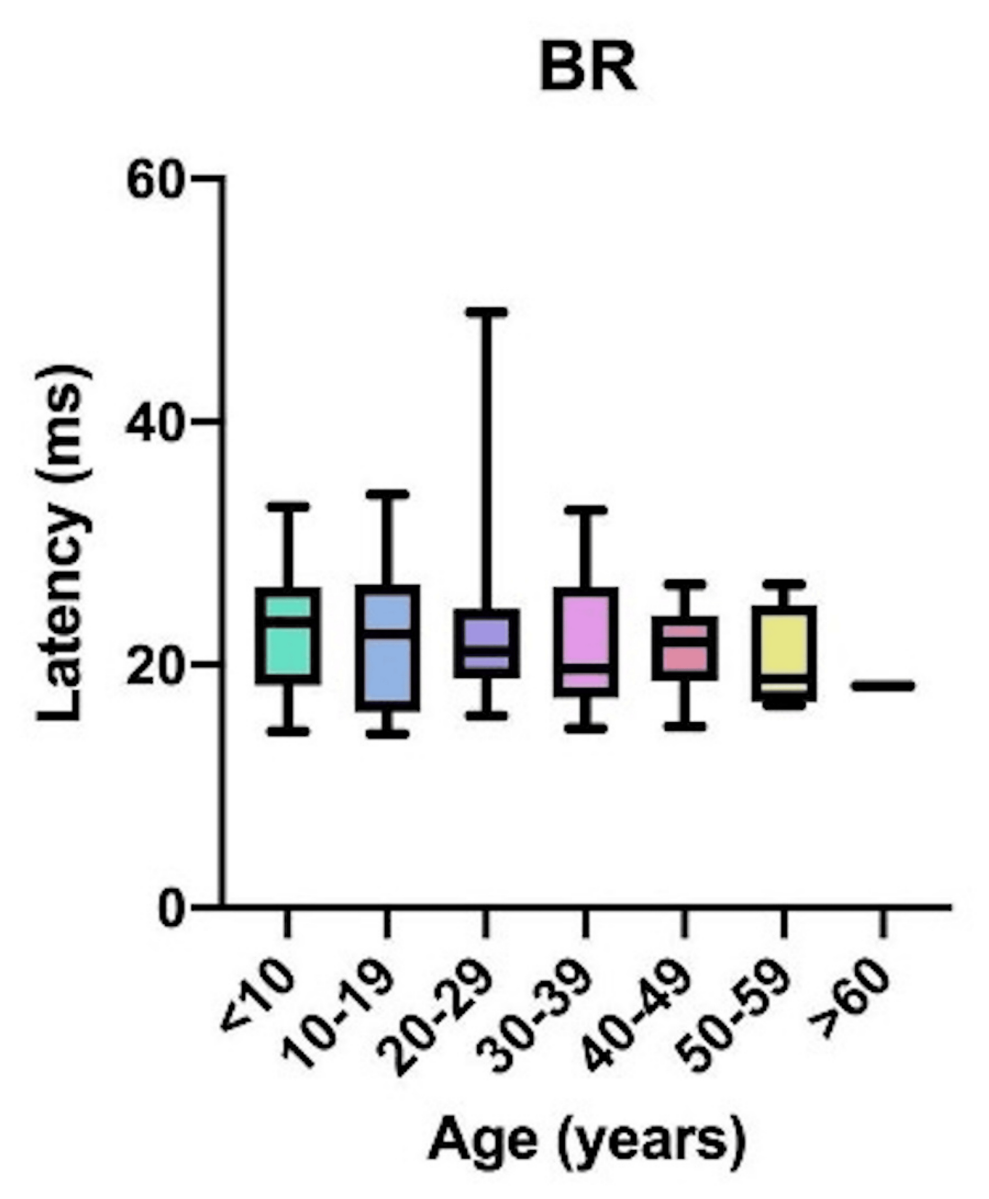
Comparison of latency for brachioradialis (BR) among different age groups

**Figure 5:**
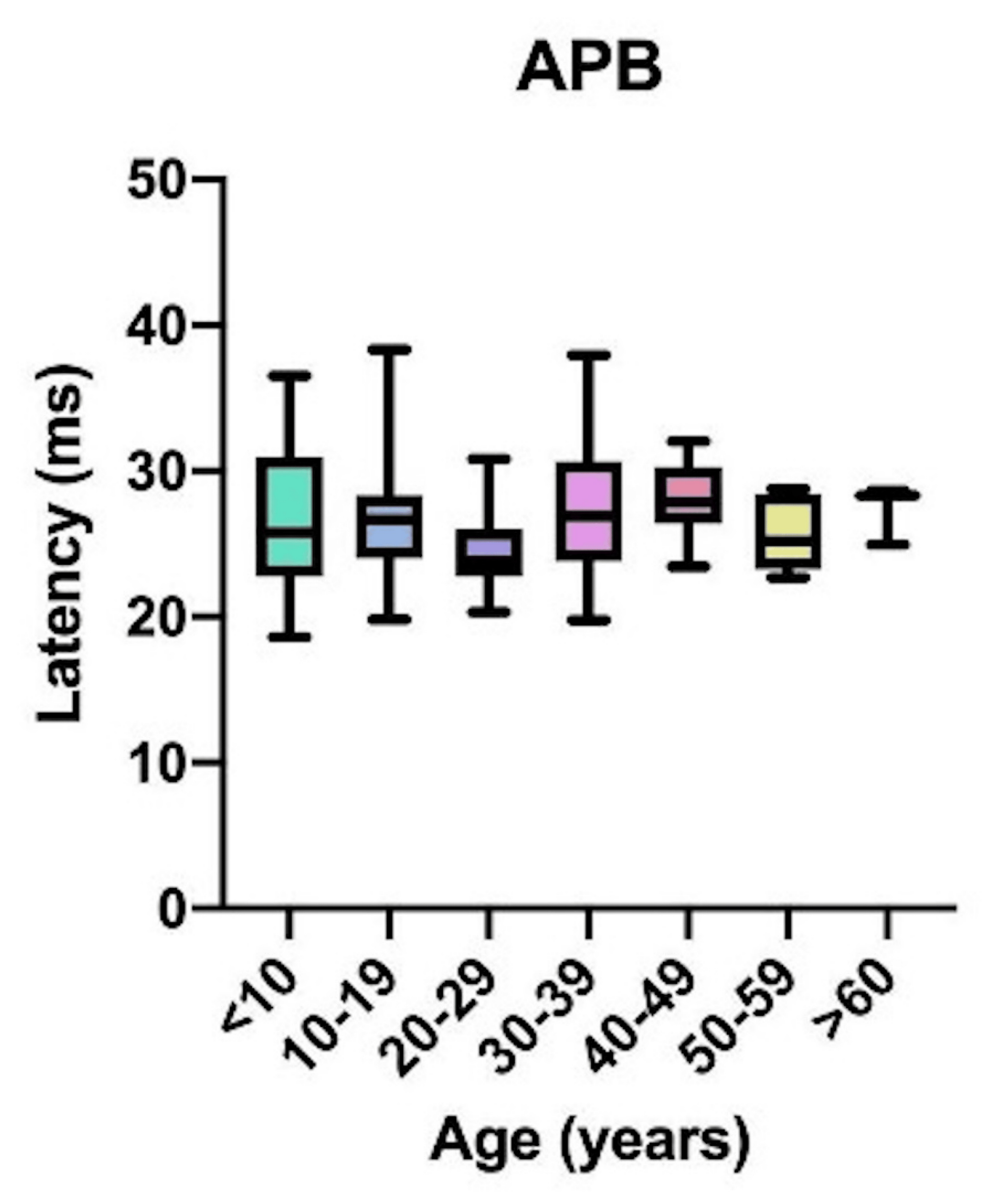
Comparison of latency for abductor pollicis brevis (APB) among different age groups

**Figure 6:**
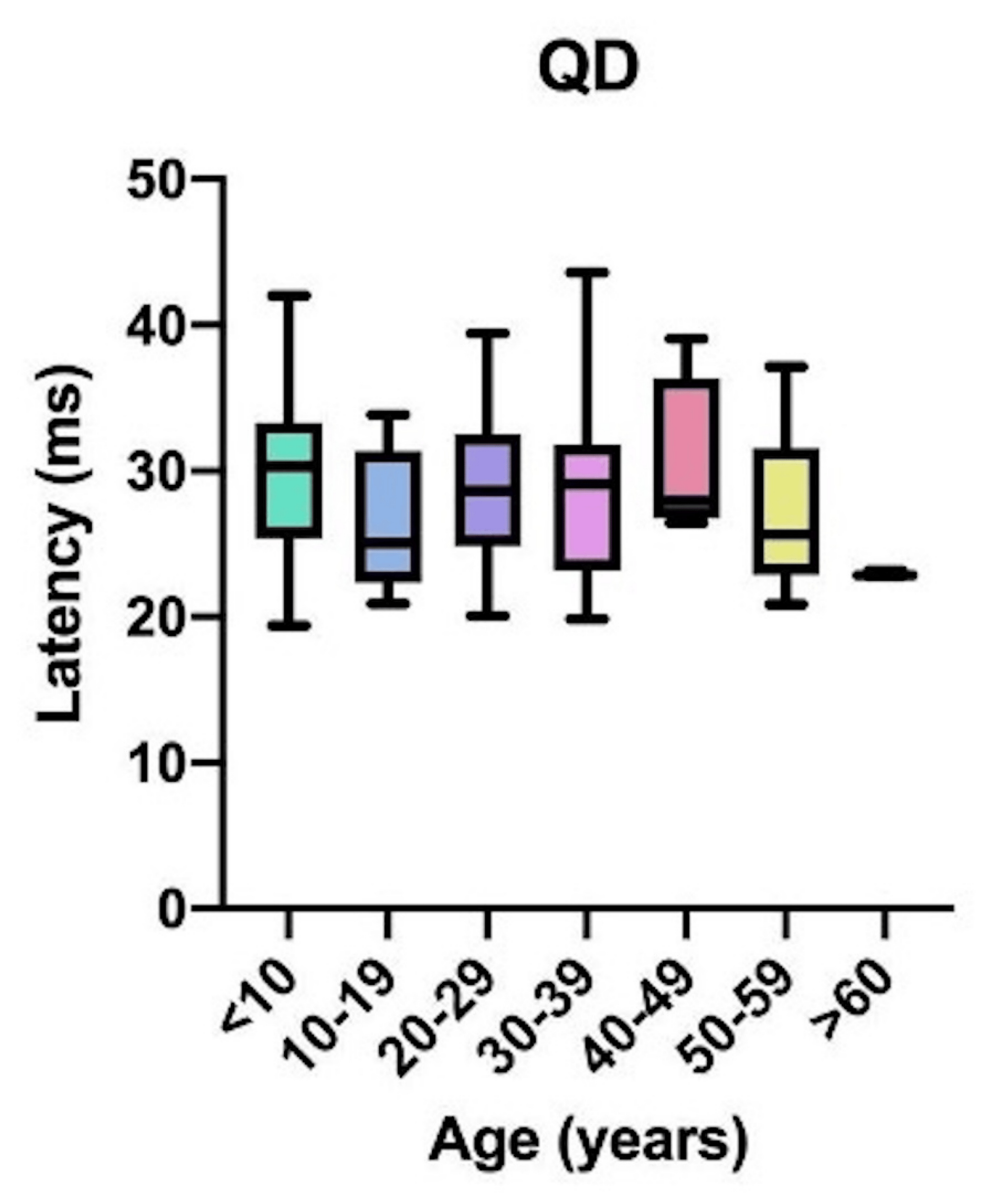
Comparison of latency for quadriceps (QD) among different age groups

**Figure 7:**
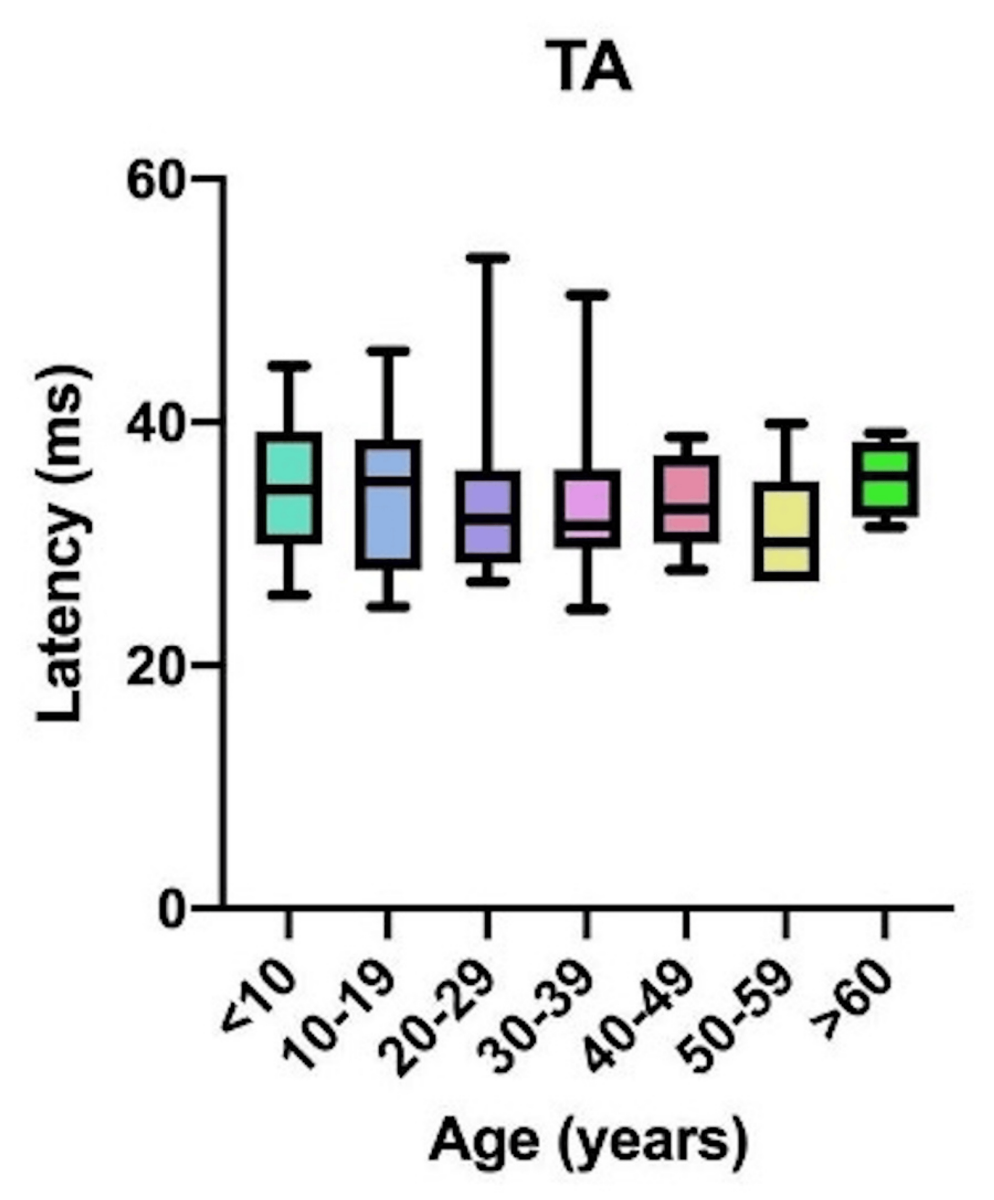
Comparison of latency for tibialis anterior (TA) among different age groups

**Figure 8:**
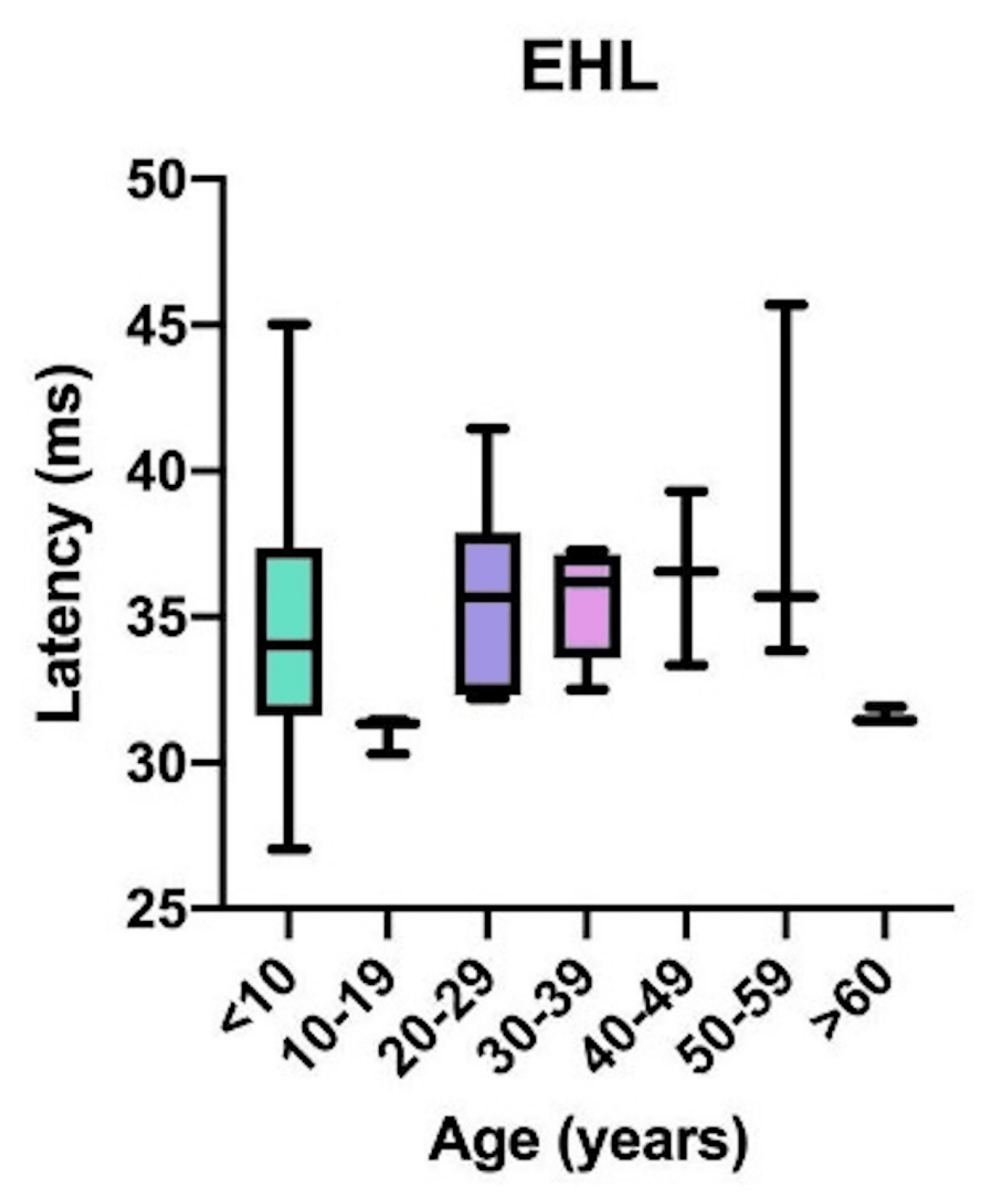
Comparison of latency for extensor hallucis longus (EHL) among different age groups

**Figure 9:**
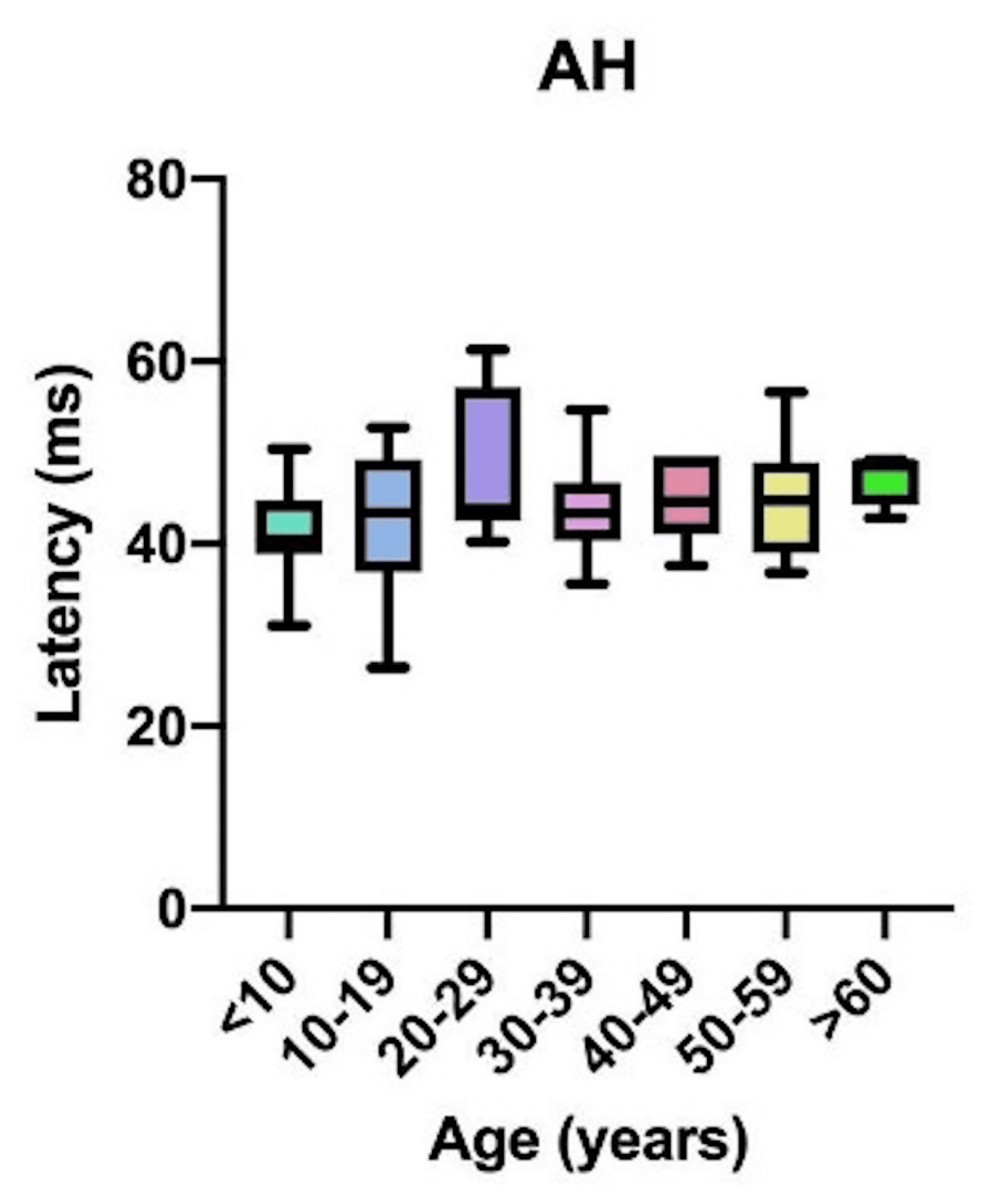
Comparison of latency for abductor hallucis (AH) among different age groups

**Figure 10:**
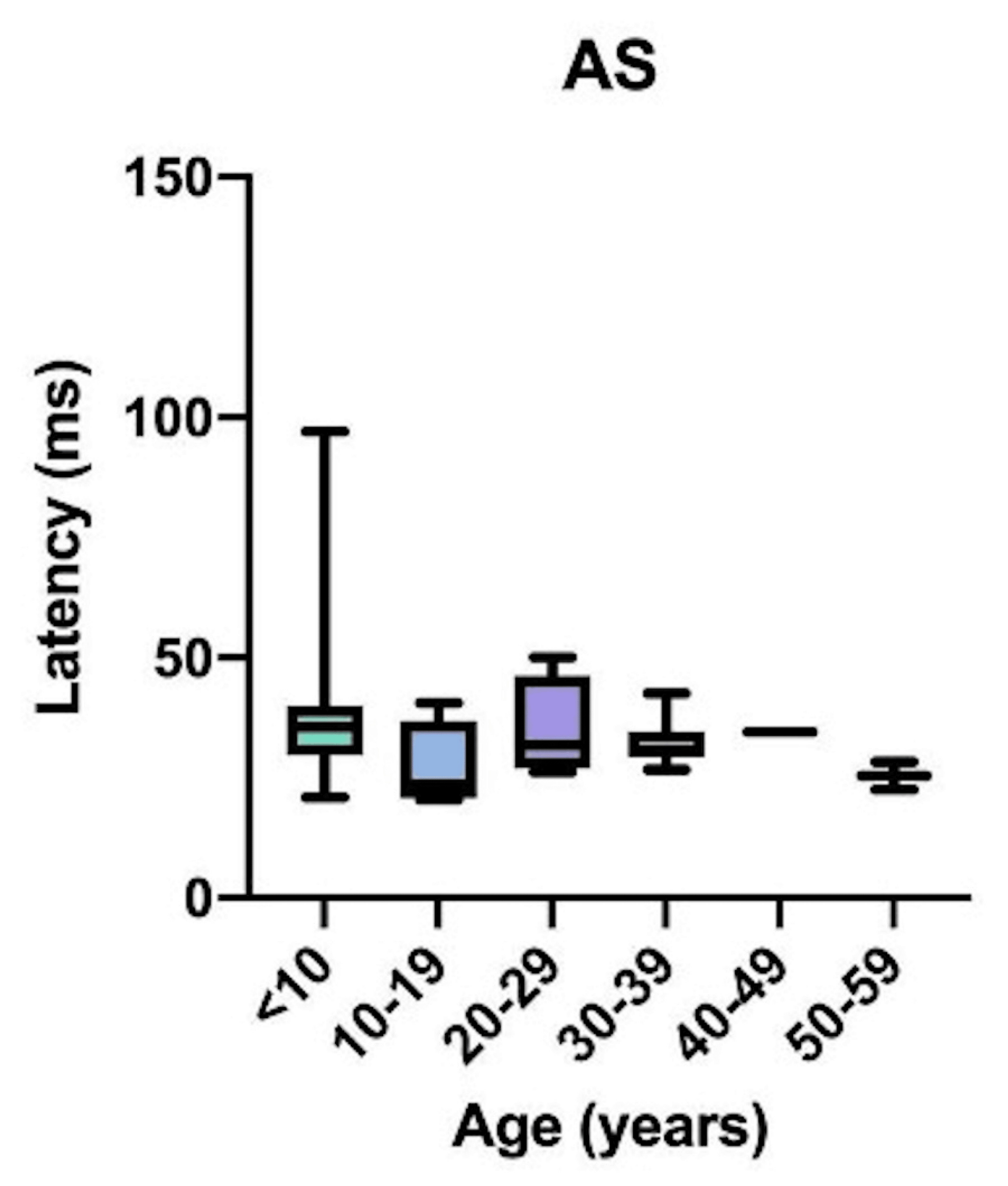
Comparison of latency for the anal sphincter (AS) among different age groups (data for age group >60 years was not available for the analyzed set of patient data)

Comparative latency data for individual muscles across age groups are presented in Figure 4: brachioradialis (BR); Figure 5: abductor pollicis brevis (APB); Figure 6: quadriceps (QD); Figure 7: tibialis anterior (TA); Figure 8: extensor hallucis longus (EHL); Figure 9: abductor hallucis (AH); and Figure 10: anal sphincter (AS).

**Figure 11:**
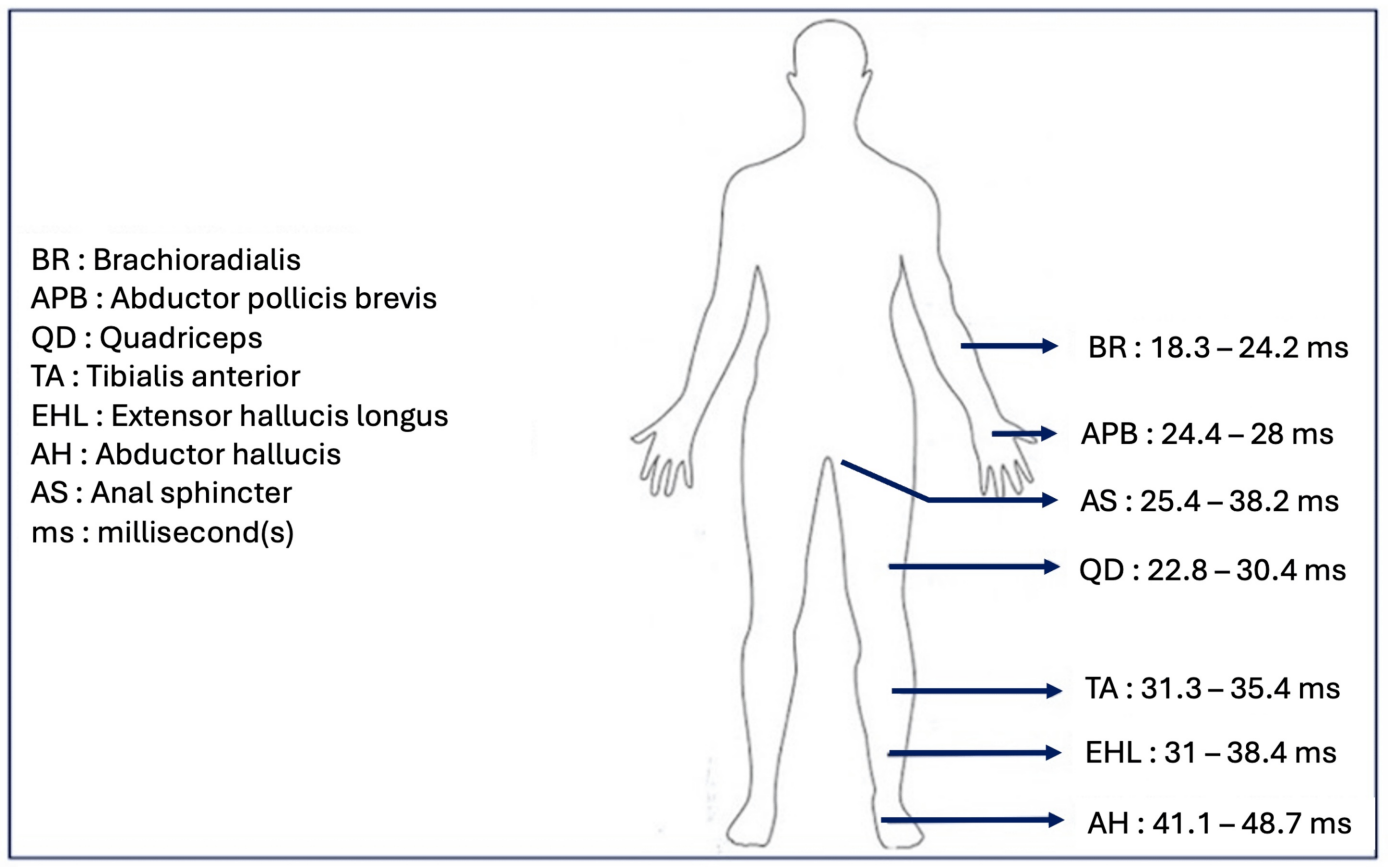
Range of latency (in milliseconds) for all ages This figure was created by the authors of this article.

Figure 11 illustrates the range of average latencies across all age groups.

Overall, age did not significantly affect MEP response latency across the studied muscles.

## Discussion

This study aimed to investigate how age affects latency in motor conduction using TcMEPs during surgery. Methods like electrodiagnostic nerve conduction studies (NCV) (Rivner et al. [6]) examined the influence of age and height on peripheral nerve conduction by relating amplitude, NCV, and distal latency to age, height, and both combined. They observed that nerve conduction velocity was negatively correlated with age in the sural, peroneal motor, and ulnar motor nerves. A smaller positive correlation was seen for the distal latencies of these nerves. Their results show that age accounts for less than 10% of the variability of NCV and distal latencies. Similar studies for normal values of Central Motor Conduction Time (CMCT), such as by Booth et al. [7], using transcranial magnetic stimulation (TMS) have been unclear as to whether these values of CMCT can be adapted to different age groups. Imajo et al. have quantified the latencies and demonstrated the effect of age using F-waves, but only for two muscles, abductor digiti minimi (ADM) and abductor hallucis (AH) [8]. Our results reveal that latency remains consistent across different age groups.

Despite physiological changes associated with growth and aging, the nervous system must compensate for changes by adjusting conduction velocity to maintain stable latency, with the plausible explanation that, as individuals grow taller, usually until 20 years of age, and experience an increase in body length, the speed of neural signal conduction also increases. Essentially, the nervous system appears to employ a compensatory mechanism that maintains efficient communication between the brain and muscles as the body undergoes physical changes. This adaptation is crucial for preserving the timing and coordination of motor functions, which are essential for smooth and effective movement. The study highlights the adaptability of the nervous system to be able to preserve and regulate precise neural timing, thereby ensuring optimal motor coordination. This adaptability allows for the maintenance of coordinated responses throughout development, without necessitating the unlearning and relearning of coordination skills or timing mechanisms. Shadmehr et al. observed that during the early years of growth, with an increase in height of the individual, it is the velocity of nerve conduction that increases without compromising on latency times [9]. This leads us to consider that the preservation of stable latencies could have a role in motor learning, sequence learning, and motor coordination.

Motor learning encompasses the modifications in an individual's movement patterns that reflect underlying changes in the nervous system's structure and function as evidenced by Krakauer et al. [10]. This process occurs across diverse timescales and levels of complexity. For instance, acquiring skills such as walking or speaking may take years, but individuals continue to adapt to alterations in physical attributes like height, weight, and strength throughout their lives. Motor learning is considered "relatively permanent" because it involves the acquisition and retention of the ability to respond appropriately to various stimuli as shown by Hikosaka et al. [11].

At the musculoskeletal level, motor learning is associated with motor units comprising motor neurons and the muscle fibers they innervate. Successful execution of even simple motor tasks requires the coordinated activity of thousands of these motor units. The body appears to manage this complexity by organizing motor units into functional modules, where the activity of these units is correlated to streamline movement.

Sequence learning is a fundamental aspect of human ability, integrated into both conscious and subconscious learning processes and activities. Sequences, whether of information or actions, play a crucial role in various daily tasks, as demonstrated by Miller et al., Miall et al., Schneider et al., Squire et al., and Schiffer et al. [12-16].

Motor coordination is the combination of body movements created with the kinematic (such as spatial direction) and kinetic (force) parameters that result in intended actions. Motor coordination is achieved when subsequent parts of the same movement, or the movements of several limbs or body parts are combined in a manner that is well-timed, smooth, and efficient with respect to the intended goal. This involves the integration of proprioceptive information detailing the position and movement of the musculoskeletal system with the neural processes in the brain and spinal cord, which control, plan, and relay motor commands as evidenced by Gandolfo et al., Schmidt et al., Sainburg et al., Proske et al., Bastian AJ. [17-21].

Without maintenance of stable latencies, an increase in height with growing age would necessitate constant re-programming or re-calibration of neural processes for maintenance of previously learned motor skills. Our study has demonstrated the stability of latencies across age groups, with the adaptation strategy being a proportional increase in conduction velocity to counterbalance the structural and anatomical changes that occur with growth. Since the number of nerve fibers remains constant, this increased conduction velocity ensures that timing, latencies, and sequencing of events for coordinated movement remain stable, thereby avoiding the need for adjustments in neural strategies as worked upon by Salthouse, Feldman et al., Desmedt et al., and Schneider et al. [1-3,14].

Our study, which divided subjects into age groups to monitor MEP response latency across different muscles, revealed uniformity across all age groups. Despite the expected variations in height and length during growth and development during the early years (up to 20 years of age), the average latencies of MEP responses remained strikingly similar. This observation suggests that the timing of neural responses remains stable even as physical dimensions change, highlighting a key aspect of motor conduction: the maintenance of latency.

This consistency in latency despite expected physical growth is also supported by foundational research. For example, Swärd et al. investigated myelinated axons in cats and documented significant changes in nodal and internodal diameters during pre and post-natal development [4]. Similarly, Raminsky et al. examined conduction properties and membrane currents in rats, observing that nodal constriction at the onset of myelination, which approached adult values over time, indicated an increase in conduction velocity [5]. Their findings also highlighted a steady increase in the internodal length of large myelinated fibers and a decrease in internodal conduction time. Bertram et al. extended these observations to human sural nerves, finding that nodal and internodal diameters reached adult values by the age of 4-5 years, with a consistent ratio between internodal and paranodal diameters [22]. These results reinforce the concept that as the nervous system matures, structural changes support increased conduction velocity while latencies remain constant.

Overall, our study underscores the nervous system's remarkable ability to maintain consistent latency and adapt its conduction velocity to accommodate changes in bodily dimensions. This adaptability ensures uninterrupted motor performance and coordination, demonstrating the system's flexibility and efficiency in supporting complex motor functions throughout development.

Strengths and future directions

This study provides valuable insights into age-specific latencies for motor-evoked potentials across different muscle groups, which can aid in establishing baseline latencies for individuals without pathological nerve conditions and conditions affecting neurotransmission. Future research could investigate how these latency measures apply to various contexts, such as evaluating polio and related disorders or cases of ascending and descending paralysis, myelination patterns, and therapeutic interventions. Longitudinal studies could further clarify these relationships and their implications for conditions like Guillain-Barré Syndrome (GBS) and other neuropathies.

Additionally, deviations from the established latency values could indicate underlying issues, such as demyelination, neuropathy, or neurodegeneration, signaling the need for further investigation.

Limitations

The study's sample was not distributed uniformly across the various age groups, which may affect the representativeness of the findings. Additionally, the study focused on normal data only and did not include longitudinal tracking to assess changes over time. Other factors, such as spinal deformities and the presence or absence of spinal plates, were not considered and could influence latency measures.

The depth of corticospinal tract activation can vary with stimulation intensity. When stimulating at threshold intensities, the activation typically occurs at more superficial layers of the cortex. Hence, latencies at threshold stimulation were measured. When using TcMEP, it is important not to elicit recordings from the limbs ipsilateral to the cortical site of activation, colloquially known as the “crossover” response. When the "crossover" response occurs, the activation site from TcMEP stimulation is thought to be subcortical. The deeper penetration of stimulus is due to the amount of current intensity used, current density, and current spread that occurs while applying transcranial stimulation. In theory, the cortical structures at risk are being bypassed by the transcranial stimulation when the "crossover" response is present, and false negatives may occur. For this reason, the “crossover” response was used as and when possible before monitoring for surgery began, for discriminating the activation of subcortical sites vs. cortical sites. However, since there is very little data to suggest that when the “crossover” response is not present, the cortical area is still not being bypassed with transcranial stimulation, this is another limitation of the study.

Addressing these limitations in future research could provide a more comprehensive understanding of latency variations and their clinical implications.

## Conclusions

The study found that average latencies measured via transcranial motor-evoked potentials remained consistent across different muscle groups and age groups. This stability was observed even in younger age groups (<10 years and 11-20 years), despite increases in height and body length. This suggests that as individuals grow, the speed of nerve conduction increases to compensate for the greater length or height, maintaining constant latency. The observation that latency remains unchanged while conduction velocity increases during early growth highlights the critical role of conduction velocity in motor learning, sequence learning, and motor coordination.

## References

[1] Salthouse TA (2000). Aging and measures of processing speed. Biol Psychol.

[2] Control of human movement. Handbook of Brain Theory and Neural Networks.

[3] Desmedt JE, Godaux E (1977). Ballistic contractions in man: characteristic recruitment pattern of single motor units of the tibialis anterior muscle. J Physiol.

[4] Swärd C, Berthold CH, Nilsson-Remahl I, Rydmark M (1995). Axonal constriction at Ranvier’s node increases during development. Neurosci Lett.

[5] Raminsky M, Ricot PJ (1987). Conduction properties of single nerve fibers in developing rat spinal nerve roots. Brain Res.

[6] Rivner MH, Swift TR, Malik K (2001). Influence of age and height on nerve conduction. Muscle Nerve.

[7] Booth KR, Streletz LJ, Raab VE (1991). Motor evoked potentials and central motor conduction: studies of transcranial magnetic stimulation with recording from the leg. Electroencephalogr Clin Neurophysiol.

[8] Imajo Y, Kanchiku T, Suzuki H (2017). Effects of differences in age and body height on normal values of central motor conduction time determined by F-waves. J Spinal Cord Med.

[9] Shadmehr R, Wise SP (2005). The Computational Neurobiology of Reaching and Pointing. A Foundation for Motor Learning. Computational Neuroscience Series.

[10] Krakauer JW, Shadmehr R (2006). Consolidation of motor memory. Trends Neurosci.

[11] Hikosaka O, Isoda M (2010). Switching from automatic to controlled behavior: cortico-basal ganglia mechanisms. Trends Cogn Sci.

[12] Miller EK, Cohen JD (2001). An integrative theory of prefrontal cortex function. Annu Rev Neurosci.

[13] Miall RC, Robertson EM (2006). Functional imaging: is the resting brain resting?. Curr Biol.

[14] Schneider W, Shiffrin RM (1977). Controlled and automatic processing: I. Detection, search, and attention. Psychol Rev.

[15] Squire LR, Dede AJ (2015). Consolidation and the hippocampus: where are we now?. Hippocampus.

[16] Schiffer AM, Schubotz RI (2011). Caudate nucleus signals for breaches of expectation in a movement observation paradigm. Front Hum Neurosci.

[17] Gandolfo F, Mussa-Ivaldi FA, Bizzi E (1996). Motor learning by field approximation. Proc Natl Acad Sci U S A.

[18] Schmidt RA, Lee TD (2011). Motor control and learning: a behavioral emphasis (5th ed.). J Hum Kinet.

[19] Sainburg RL, Wang J (2006). The effects of central nervous system injury on motor control. Handbook of Clinical Neurology.

[20] Proske U, Gandevia SC (2012). The proprioceptive senses: their roles in signaling body shape, body position and movement, and muscle force. Physiol Rev.

[21] Bastian AJ (2006). Learning to predict the future: the cerebellum adapts feedforward movement control. Curr Opin Neurobiol.

[22] Bertram M, Schröder JM (1993). Developmental changes at the node and paranode in human sural nerves: morphometric and fine-structural evaluation. Cell Tissue Res.

